# Lipocalin-2 drives neuropsychiatric and cutaneous disease in MRL/lpr mice

**DOI:** 10.3389/fimmu.2024.1466868

**Published:** 2024-09-27

**Authors:** Sayra J. Garcia, Elise V. Mike, Jinghang Zhang, Carla M. Cuda, Chaim Putterman

**Affiliations:** ^1^ Department of Microbiology and Immunology, Albert Einstein College of Medicine, Bronx, NY, United States; ^2^ Wilmer Eye Institute, Johns Hopkins University, Baltimore, MD, United States; ^3^ Department of Medicine, Northwestern University, Chicago, IL, United States; ^4^ Azrieli Faculty of Medicine, Bar Ilan University, Zefat, Israel

**Keywords:** systemic lupus erythematosus, neuropsychiatric lupus, lipocalin-2, MRL/lpr, cutaneous systemic lupus erythematosus

## Abstract

**Introduction:**

Approximately 20-40% of patients with systemic lupus erythematosus (SLE) experience neuropsychiatric SLE (NPSLE), which often manifests as cognitive dysfunction and depression. Currently, there are no approved treatments for NPSLE because its underlying mechanisms are unclear. Identifying relevant mediators and understanding their contribution to pathogenesis are crucial for developing targeted treatment options. Lipocalin 2 (LCN2) is a multifunctional acute-phase protein that plays important roles in immune cell differentiation, migration, and function. LCN2 has been implicated in models of neuroinflammatory disease.

**Methods:**

We generated an LCN2-deficient MRL/lpr mouse to evaluate the effects of LCN2 on this classic NPSLE model. To evaluate the effects of LCN2 deficiency on behavior, the mice underwent a battery of behavioral tests evaluating depression, memory, and anxiety. Flow cytometry was used to quantify immune cell populations in the brain, blood, and secondary lymphoid organs. Cutaneous disease was quantified by scoring lesional skin, and skin infiltrates were quantified through immunofluorescent staining. Systemic disease was evaluated through measuring anti-nuclear antibodies by ELISA.

**Results:**

In this study, we found that LCN2 deficiency significantly attenuates neuropsychiatric and cutaneous disease in MRL/lpr lupus prone mice, likely by decreasing local infiltration of immune cells into the brain and skin and reducing astrocyte activation in the hippocampus. Anti-nuclear antibodies and kidney disease were not affected by LCN2.

**Discussion:**

As there was no effect on systemic disease, our results suggest that the inflammatory effects of LCN2 were localized to the skin and brain in this model. This study further establishes LCN2 as a potential target to ameliorate organ injury in SLE, including neuropsychiatric and cutaneous disease.

## 1 Introduction

Systemic lupus erythematosus (SLE) is a chronic autoimmune disease characterized by autoreactive B and T lymphocytes, the presence of anti-nuclear antibodies, and multiple clinical manifestations (1). The central nervous system (CNS) or brain involvement in lupus is referred to as neuropsychiatric SLE and affects approximately 20–40% of patients with lupus. NPSLE increases the mortality risk, decreases the quality of life, and can precede or occur independently of active systemic disease (2, 3). Targeted therapies have yet to be developed or implemented because disease pathways are still not fully understood. Potential mechanisms implicated in NPSLE include blood-brain barrier (BBB) disruption, choroid plexus (CP) pathology and dysfunction, pathogenic autoantibodies generated locally or coming from the periphery and targeting neural tissue, and neuroinflammation with the activation of microglia and astrocytes. Treatment options for patients with NPSLE remain nonspecific and focus on general immunosuppression, with prolonged glucocorticoid treatment often exacerbating depression and cognitive impairment (4).

Lipocalin-2 (LCN2) is a 25 kDa secreted acute phase protein involved in iron homeostasis and immune processes. LCN2, also known as neutrophil gelatinase-associated lipocalin, is a member of the lipocalin superfamily, a family of transporters of small hydrophobic molecules. Owing to their diverse functions, LCN2 and its receptors are located in many tissues throughout the body and several cell types in the brain. Innate immune cells, lymphocytes, and brain glial cells produce LCN2 in response to inflammation (5). Moreover, LCN2 affects both innate and adaptive immunity by promoting cell differentiation, survival, activation, and migration, suggesting that this mediator can function in an autocrine manner.

LCN2 has been implicated in several neuroinflammatory and neurodegenerative diseases including Parkinson’s disease, Alzheimer’s disease, multiple sclerosis, and ischemic stroke (6–9). Several possible effects of LCN2 may be instrumental in the pathogenesis of brain disease, including the induction of neurotoxicity, activation of astrocytes and endothelial cells (and disruption of the BBB), promotion of leukocyte chemotaxis from systemic circulation, and stimulation of cytokine and chemokine secretion (10–13). While astrocytes are an important source of brain LCN2, other brain cell types including neurons, endothelial cells, and microglia can produce LCN2 as well as express LCN2 receptors. Notably, pro-inflammatory cytokines classically associated with NPSLE, such as CXCL10, IL-6, TNF, and interferons, are known to induce LCN2 expression and secretion (14, 15).

Animal models are of particular importance in NPSLE research because tissues are rarely available from human patients with lupus with acute neuropsychiatric disease, especially prior to treatment (16). While LCN2 deficiency was previously demonstrated to have an ameliorative effect on neurobehavioral deficits exhibited by B6.SLE1.SLE3 mice (17), its effects on a more robust and representative NPSLE model have yet to be explored. MRL/lpr mice, an inbred strain with defective Fas-mediated apoptosis, mimic human lupus in the presence of autoantibodies, cutaneous disease, glomerulonephritis, lymphoproliferation, and prominent cognitive and emotional deficits. MRL/lpr mice are the most widely used murine lupus strain to explore NPSLE. In this study, we compared the behavioral phenotype of LCN2-deficient to that of LCN2-sufficient MRL/lpr mice, identified the main brain source of LCN2 in this strain, and characterized the effects of LCN2 deficiency on cell populations in the brain and periphery.

## 2 Materials and methods

### 2.1 Mice

MRL/lpr mice were purchased from Jackson Laboratories (Bar Harbor, ME, USA) or bred in-house. B6.LCN2-KO mice (18) were crossed with MRL/lpr mice for 10 generations to create MRL/lpr-LCN2-KO (LCN2-KO) mice. C57BL/6 (B6) mice were bred in-house, and MRL/MpJ mice were purchased from Jackson Laboratories. All mice were aged or bred at the Albert Einstein College of Medicine Animal Facility (Bronx, NY, USA) and had ad libitum access to water and food. The mice received the standard laboratory rodent diet 5001 formula, with a composition as follows: crude protein: 23%, crude fat: 4.5%, crude fiber: 6%, and ash: 8%.

All mice in these studies were female and housed on a 12:12 h light:dark cycle at a temperature of 21–23°C. All animal husbandry and protocols were approved by the Institutional Animal Care and Use Committee of the Albert Einstein College of Medicine (protocol #00001201).

### 2.2 Behavioral assessment

Mice undergoing behavioral testing were assessed for general locomotor function and activity using a behavioral spectrometer. Cognitive functions (spatial and recognition memory) were assessed using object placement (OP) and object recognition (OR) tests (19, 20). Depression-like behavior was tested using the Porsolt swim and tail suspension tests (21). The mice were acclimated to the testing room for at least 30 min before the tests began.

#### 2.2.1 Behavioral spectrometry

Due to illness and presence of lymphadenopathy in MRL/lpr mice, movement and general activity limits can be confounding factors in subsequent behavioral tests evaluating the neuropsychiatric disease state. The behavioral spectrometer (Behavioral Instruments, Hillsborough, NJ, USA) is a 40 cm^2^ enclosed arena equipped with a vibration-sensitive floor and a video tracking system that can detect and quantify behavior (Viewer software, Biobserve, Berlin, Germany). The mice were placed in the arena for a total of 9 min, and the software quantified general locomotion (track length), time spent active or still, and behaviors, such as grooming or rearing (22). The mice were excluded from subsequent behavioral tests if insufficient activity was observed.

#### 2.2.2 Cognitive function assessment - OR and OP tests

The OP and OR tests both rely on the natural preference of mice for novel objects. When given a choice, cognitively preserved mice exhibit a preference for exploring novel objects, as opposed to objects to which they have been previously exposed. To test for intact memory, the preference for a novel position or object is measured; if the mice recognize that the test object is novel or in a new position, they will spend more time with that object than with the unchanged object. Both the OR and OP tests require two trials to assess cognitive function. The first was a training trial. Mice were placed in an arena with two identical objects and given a set amount of time to explore the objects before they were removed for a retention period. After the set retention interval, the mice were returned to the arena for testing. For the OR test, one of the objects in the training arena was replaced with a different but similarly stimulating object. In the OP test, one of the objects was moved to a different location within the arena. The replaced or moved object was deemed a novel object. A stopwatch was used to record the amount of time the mice spent with each object in both tests. Preference scores (%) were calculated as the percentage of time spent on novel objects. A passing score was considered any score ≥55%, as previously determined as the passing score cutoff for this strain (17, 21, 23).

For the OR task, the mice were trained for 4 min with a retention interval of 60 min and a testing time of 4 min. For the OP task, mice were trained for 5 min, the retention interval was 40 min, and the testing time was 4 min. Abnormally behaving mice (i.e., insufficient time to explore objects during the training period, spinning, or not moving within the arena) were excluded from the trial. Each trial was recorded using Viewer software (19, 20).

#### 2.2.3 Depression-like behavior assessment - Porsolt swim test

Mice were placed in a clear beaker of 27°C water for 10 min. The first minute was not scored to allow the mice to acclimate to the water, after which they were scored manually for 9 min. The mice were assessed for immobility, which was defined as the lack of movement in at least three of the five limbs (four paws and the tail). Percent immobility was calculated as the total time spent immobile divided by the total time (9 min). Each trial was recorded using Viewer software (17, 21, 23, 24).

#### 2.2.4 Depression-like behavior assessment - tail suspension test

Mice were suspended in a 24” × 24” tail suspension box. The first minute was not scored to allow the mice to acclimate to the position, after which they were manually timed for immobility for over 3 min. Percent immobility was calculated as the total time spent immobile divided by the total time (3 min). Each trial was recorded using Viewer software (24).

### 2.3 Assessment of systemic and renal disease

To monitor kidney involvement, urine was semi-quantitatively tested for proteinuria using Uristix (Siemens Healthcare Diagnostics, Tarrytown, NY, USA). Systemic disease was evaluated by quantifying serum anti-nuclear antibodies and circulating peripheral blood mononuclear cells (PBMCs), as previously described (25, 26).

### 2.4 Enzyme-linked immunosorbent assays

Circulating IgG, anti-dsDNA, anti-histone, and anti-chromatin antibodies were measured using ELISA with serum samples collected at the time of sacrifice, as previously described (24, 25).

### 2.5 Assessment of cutaneous disease

Macroscopic skin lesions were blindly scored by trained observers immediately before sacrifice. Numerical values based on erythema, alopecia, scaling, and skin thickening were assigned to multiple body regions including the body, face, and limbs. The scores were adjusted for the degree of severity and the percentage of body surface area covered (27).

### 2.6 Immunofluorescence staining

At the time of sacrifice, mice were intracardially perfused with ice-cold PBS. Lesional skin and brains were harvested, fixed in 4% paraformaldehyde (PFA), and stored in 2.5% PFA for 3 days. Tissues were embedded in paraffin and sectioned. Sections were deparaffinized in xylene, rehydrated, and boiled in citrate buffer (pH 6) for 5 min for antigen retrieval. Tissues were blocked with 20% horse serum. For the antibody cocktails, the antibodies were diluted in 2% horse serum and 0.01% triton. An EVOS FL Auto 2 automated fluorescence microscope (Thermo Fisher Scientific, Waltham, MA, USA) was used to image the slides. Images were analyzed by either manually counting stained cells, or measuring mean fluorescence intensity using ImageJ analysis software (28–30).

The antibodies used for staining skin and brains were as follows:

- Astrocytes (GFAP): primary antibody of rat anti-mouse GFAP IgG (1:100, Abcam, Waltham, MA, USA), followed by a secondary antibody of Alexa-Fluor 488 conjugated donkey anti-rat IgG (1:100, Jackson ImmunoResearch Laboratories, West Grove, PA, USA).
- C3: Primary antibody of goat anti-mouse C3 IgG (1:100, MP Biomedicals, Santa Ana, CA, USA), followed by secondary antibody of Alexa-Fluor 594 conjugated donkey anti-goat IgG (1:100, Jackson ImmunoResearch Laboratories).
- IgG: Primary and secondary antibodies Alexa-Fluor 488 conjugated donkey anti-mouse IgG (1:300).
- LCN2: Primary antibody of goat anti-mouse LCN2 IgG (1:100, R&D Systems, Minneapolis, MN, USA), followed by secondary antibody of Alexa-Fluor 594 conjugated donkey anti-goat IgG (1:100; Jackson ImmunoResearch Laboratories).
- Macrophages/microglia (Iba1): Primary antibody of rabbit anti-mouse Iba1 IgG (1:250, Wako, Richmond, VA, USA), followed by secondary antibody of Alexa-Fluor 647 conjugated donkey anti-rabbit IgG (1:100, Jackson ImmunoResearch Laboratories).
- Neutrophils (Ly6G): Primary antibody of goat anti-mouse Ly6G IgG (1:100, BD), followed by secondary antibody of Alexa-Fluor 647 conjugated donkey anti-goat IgG (1:100, Jackson ImmunoResearch Laboratories).
- T cells (CD3): Primary antibody of rabbit anti-mouse CD3eIgG (1:100, Invitrogen, Waltham, MA, USA), followed by secondary antibody of Alexa-Fluor 594 conjugated donkey anti-rabbit IgG (1:100, Jackson ImmunoResearch Laboratories).

### 2.7 Flow cytometry

#### 2.7.1 Brain single cell suspension preparation

Single cell suspensions from brain were prepared as previously described (31). Briefly, mice were transcardially perfused with ice-cold Hank’s balanced salt solution (HBSS) containing Ca^2+^ and Mg^2+^. The brains were removed and stored in HBSS on ice. Brains were infused with digestion buffer (13 w/U Liberase TL (Roche) and 1 mg/mL DNase I (Roche) in HBSS with Ca^2+^ and Mg^2+^), then placed into tubes where they were physically dissociated with clean scissors and incubated with inversion at 37°C for 45 min. The digested tissue was then passed through a 40 µM nylon cell strainer, followed by a wash of 50 mL wash buffer (4% FBS in HBSS with Ca^2+^ and Mg^2+^). Cells were spun at 1012 rcf for 15 min at 4°C, and the supernatant was discarded. The cell pellets were then suspended in 10 mL of 30% Percoll solution (30% Percoll in HBSS). Ten milliliters of the cell-Percoll suspension was then layered onto 2 mL of 70% Percoll solution in HBSS and centrifuged for 30 min at 600 rcf. The 70–30% interphase layer was collected, diluted three-fold with HBSS, and centrifuged for 20 min at 980 rcf at 20°C. The supernatant was discarded and the pellet was transferred to a round-bottom flow tube, washed with wash buffer, and plated on a 96-well V-plate before staining.

#### 2.7.2 Spleen, PBMC, and lymph node single-cell suspension preparation

Before sacrifice, 200 µL of blood was collected retro-orbitally. Mice were transcardially perfused with ice-cold HBSS containing Ca^2+^ and Mg^2+^. Spleens and cervical lymph nodes were dissected and placed into ice cold wash buffer, and passed through a 40-µm cell strainer followed by 5 mL of wash buffer. Red blood cells were lysed using ACK lysis buffer (Thermo Fisher Scientific) on ice for 10 min. After red blood cell lysis, the cells were plated onto a 96-well V-plate for staining.

#### 2.7.3 Staining

Cells were incubated with a fixable viability dye (Biolegend, San Diego, CA, USA) in PBS for 20 min. Before incubation with the conjugated antibodies, the cells were blocked with an anti-mouse CD16/32 antibody (BioLegend, clone S17011E) in a staining buffer (3% FBS in PBS). For intracellular staining, the cells were fixed, permeabilized, and stained using the eBioscience Foxp3/Transcription Factor Staining Buffer Set (Invitrogen) according to the manufacturer’s instructions and fluorochrome-conjugated intracellular antibodies. The cells were analyzed using an Aurora full spectrum analyzer (Cytek Biosciences) and FlowJo V10. The following dye-conjugated antibodies were purchased from Biolegend: Ly6C-Pacific Blue (clone HK1.4), Cd11c-BV510 (clone N418), TNFalpha-BV605 (clone MP6-XT22), CD196-BV785 (clone 29-2L17), Ly6G-PerCP (clone 1A8), CD30-PE (clone mCD30.1), B220-PE/Fire 700 (clone RA3-6B2), CD194-PE-Cy7 (clone 2G12), IL6-APC (clone MP5-20F3), and NK1.1-APC/Fire 810 (clone S17016D). CD8-53-6.7 (clone 53-6.7), MHCII-BUV496 (clone M5/114.15.2), CD19-BUV563 (clone D3), CD4-BUV737 (clone GK1.5), Cd11b-BUV805 (clone M1/70), CD56-BV 421 (clone 809220), IL17-BV650 (clone THC11-18H10), IFNg-BV750 (clone XMG1.2), CD138-BB15 (clone 281-2), and IL10-R718 (clone JES5-16E3) were purchased from BD Biosciences. CD45-Alexa Fluor 350 (clone 30-F11) was purchased from R&D Systems. F4/80-Star Bright SBV570 (clone A3-1) was purchased from Bio-Rad. FoxP3-PerCP-eFluor 710 (clone FJK-16s) was purchased from Thermo Fisher Scientific (**Supplementary Table 1**).

### 2.8 Primary astrocyte cultures

Cortical astrocytes were isolated from P1-P3 MRL/lpr pups, as described (32). The brains were dissected, minced in ice-cold HBSS, and placed in 0.025% Trypsin in EDTA at 37°C for 5 min for chemical dissociation. Trypsin was neutralized using cell suspension medium (10% FBS and 1% penicillin-streptomycin in DMEM), and the cells were further dissociated by trituration using a pipette. Cells were then centrifuged at 37°C at 1012 rcf for 5 min, the supernatant was aspirated, and the pelleted cells were plated in cell suspension media.

For exposure to different treatment conditions, the cells were plated at a density of 2 × 10^5^ cells on poly-d-lysine coated 8 mm glass coverslips in 24-well tissue culture plates. Twelve h after plating, thecell suspension medium was replaced with serum-free medium (1% penicillin-streptomycin in phenol red-free DMEM). After 1 h of starvation, cells were treated with either 100 ng/mL lipopolysaccharide (LPS; Thermo Fisher Scientific), 25 µg/mL of LCN2 (R&D Systems, Minneapolis, MN, USA), or 1 µg/mL of interferon-gamma. After 12 h, the medium was aspirated, and the cells were rinsed and fixed in 4% PFA for 5 min. Cells were incubated with an antibody cocktail of rabbit anti-mouse S100B IgG (1:250, Abcam), rat anti-mouse GFAP IgG (1:500, Invitrogen), or goat anti-mouse C3 IgG (1:100) in 4°C overnight. Cells were rinsed and incubated with Alexa-Fluor 594 conjugated donkey anti-goat IgG, Alexa-Fluor 488 conjugated donkey anti-rat IgG, and Alexa-Fluor 647 conjugated donkey anti-rabbit IgG antibodies (1:100, Jackson ImmunoResearch Laboratories) in PBS for 1 h. The coverslips were mounted on slides using Fluoromount (Thermo Fisher Scientific) and imaged using a Leica Sp8 confocal microscope.

### 2.9 Statistical analysis

Statistical analyses were performed using the GraphPad Prism software. For the analysis of MRL/lpr and LCN2-KO mice, both groups were assessed for normality and outliers (Grubb’s outlier test). Normal distribution was determined using the Shapiro–Wilk test. For experiments comparing LCN2-KO mice with MRL/lpr mice only, the Student’s t-test was used to compare normally distributed groups, and the Mann–Whitney test was used to compare nonparametric data. In experiments involving MpJ mice, a one-way ANOVA was used to compare the three groups. For all figures, *p<0.05, **p<0.01, ***p<0.001, and ****p<0.0001.

## 3 Results

### 3.1 LCN2 deficiency improves spatial memory impairment and depression-like behavior in MRL/lpr mice

**Figure 1:**
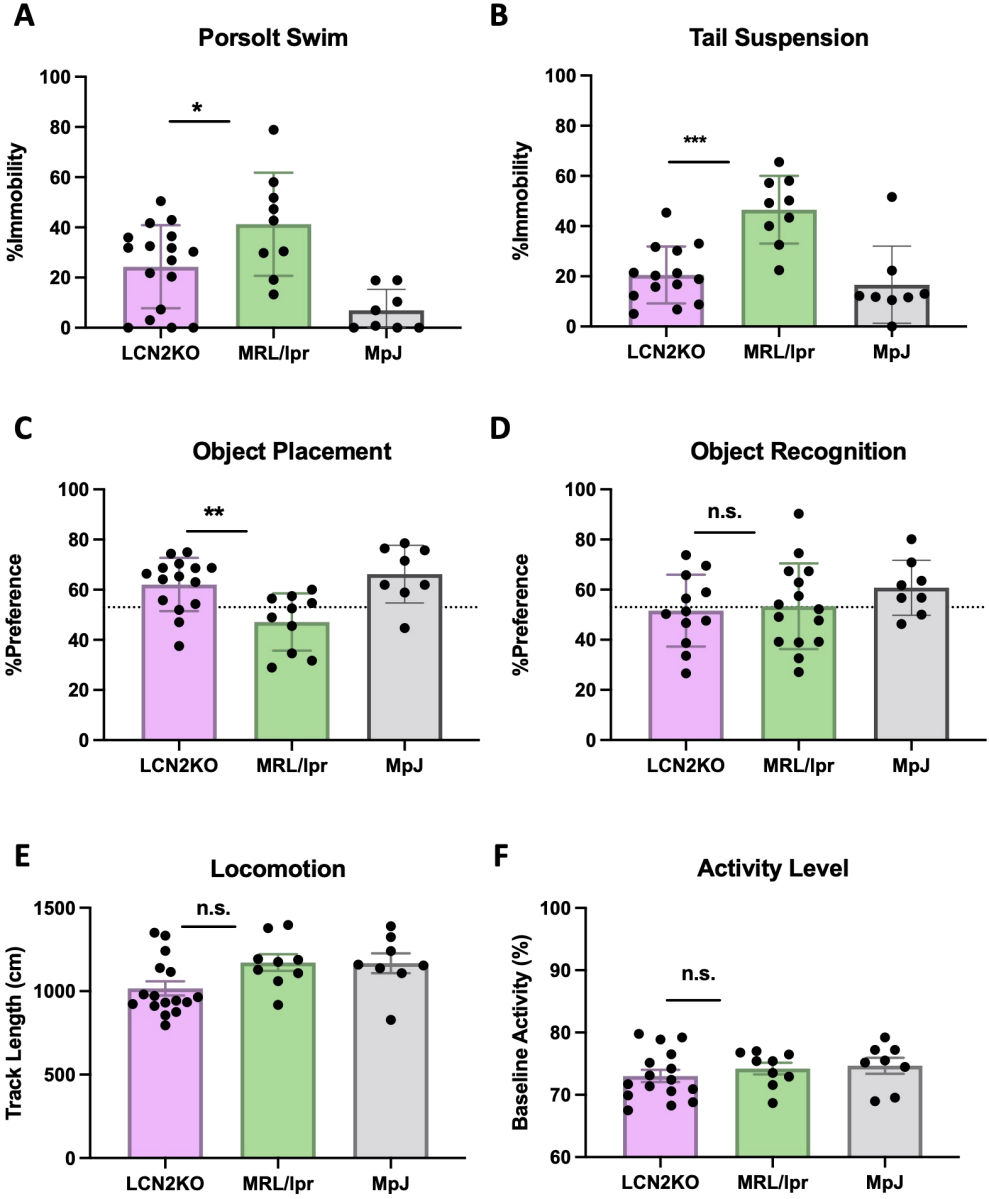
LCN2 deficiency improves behavioral abnormalities in MRL/lpr mice. **(A, B)** LCN2 deficient mice showed significant improvement in depression-like behavior in the Porsolt swim and the tail suspension tests. **(C)** LCN2-deficient mice showed improvement in spatial memory during the object placement task. **(D)** Pattern recognition memory was unaffected in LCN2-deficient mice as displayed in the object recognition task. **(E)** Locomotion was not different between groups as quantified through the behavioral spectrometer. **(F)** Behavioral spectrometry showed no differences in generalized activity between the strains. For all panels: n.s., not significant; *p<0.05, **p<0.01, and ***p<0.001.

We derived MRL/lpr and MRL/lpr LCN2-KO mice as described in the Materials and Methods and **Supplementary Material**. To evaluate whether LCN2 deficiency improves neuropsychiatric symptoms, we performed neurobehavioral assessments in MRL/lpr and MRL/lpr LCN2-KO mice between 16 and 18 weeks of age. Porsolt swim and tail suspension tests are commonly used to evaluate depression-like behavior in rodents. In the Porsolt swim test, mice were placed in water and immobility was measured. More severe behavioral despair is indicated by a higher percentage of time spent immobile instead of actively trying to swim during the test. We found that MRL/lpr LCN2-KO mice spent significantly less time immobile than MRL/lpr mice (LCN2-KO (n = 16) vs MRL/lpr (n = 9), 24.32 ± 4.01 vs 41.26 ± 6.84, p=0.04, **Figure 1A**). Similarly, MRL/lpr LCN2-KO mice had significantly less overall immobility in the tail-suspension test compared to the LCN2 wild-type MRL/lpr mice (LCN2-KO (n = 16) vs MRL/lpr (n = 9), 20.55 ± 3.03 vs 46.51 ± 4.50, p = 0.0002, **Figure 1B**). These results suggest that LCN2 deficiency improves depression-like behaviors in MRL/lpr mice.

Cognition, in the form of spatial and pattern recognition memory, were examined in the two experimental strains by the standard OP and OR tasks. In the OP task, which tests for spatial memory, 12 out of 15 (80%) MRL/lpr LCN2-KO mice passed, as compared with 6 of the 18 (33%) MRL/lpr mice (LCN2KO (n = 16) vs MRL/lpr (n = 9), 62.08 ± 2.74 vs 47.82 ± 2.87, p = 0.003, **Figure 1C**). In the OR task, which tests for pattern recognition memory, 5 of the 12 (41.6%) MRL/lpr LCN2-KO mice passed, as compared to 7 of the 15 (46.7%) MRL/lpr mice (LCN2-KO (n = 16) vs MRL/lpr (n = 9), 51.63 ± 4.128 vs 53.35 ± 4.41, p = 0.91, **Figure 1D**). These results indicate that LCN2 deficiency improves spatial, but not pattern recognition memory impairments.

The swim and memory tests are physically demanding; therefore, we ensured that the observed differences were not due to physical impairment by evaluating locomotion and baseline activity. We used a behavioral spectrometer to measure track length and baseline activity levels. LCN2-KO and MRL/lpr mice showed similar activity levels in locomotion (LCN2-KO (n = 16) vs MRL/lpr (n = 9), 71.53 ± 0.97 vs 74.72± 0.98, p = 0.09, **Figure 1E**) and baseline activity (LCN2-KO (n = 16) vs MRL/lpr (n = 9), 989.00 ± 46.87 vs 1175.00 ± 44.41, p = 0.12, **Figure 1F**), confirming that the observed phenotypes in the OP, forced swim, and tail suspension tests were not due to impaired mobility or sickness behavior.

### 3.2 LCN2 deficiency decreases immune cell infiltrates but does not affect immune deposition or barrier function in MRL/lpr mice

**Figure 2:**
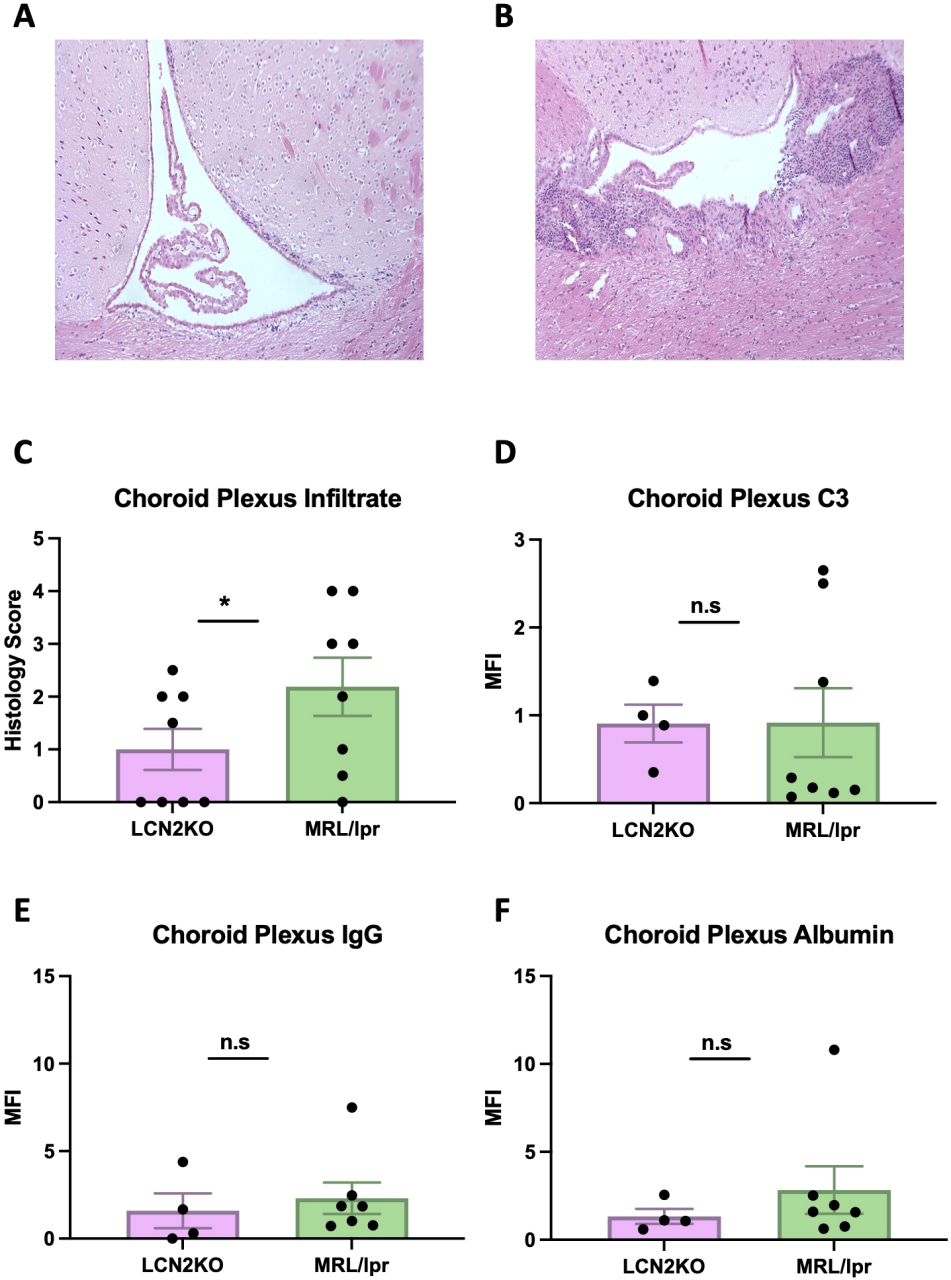
LCN2 deficiency decreases CP infiltrates. **(A)** Representative H&E image of CP infiltration in an LCN2-KO mouse. **(B)** Representative H&E image of the CP in an MRL/lpr mouse. **(C)** LCN2-KO mice have lower CP infiltrates when scored quantitatively. **(D)** CP C3 and **(E)** IgG expression were unaffected by LCN2 deficiency. **(F)** Albumin leakage in CP was unchanged in LCN2-deficient mice compared to MRL/lpr mice. For all panels: n.s., not significant and *p<0.05.

As there was significant attenuation of the neuropsychiatric phenotype in LCN2-KO MRL/lpr mice compared to that in LCN2 wild-type mice, we determined the mechanism of protection. LCN2 plays diverse roles in immune cell regulation and BBB integrity in neuroinflammatory diseases; therefore, we assessed the permeability of the BBB and blood-cerebrospinal barrier by examining immune cell infiltration in the CP and quantifying albumin, C3, and IgG deposition in several relevant brain regions (cortex, hippocampus, and CP). The MRL/lpr strain is characterized by extensive lymphocytic infiltration of the CP (21, 30). We found that CP infiltration was decreased in LCN2-KO mice at the light microscopy level (LCN2-KO (n = 8) vs MRL/lpr (n = 8), 1.00 ± 0.39 vs 2.19 ± 0.55, p = 0.05, **Figures 2A–C**). C3 deposition was minimally reduced in the CP of MRL/lpr LCN2-KO mice compared with that in MRL/lpr mice (LCN2-KO (n = 4) vs MRL/lpr (n = 8), 0.88 ± 0.21 vs 0.92 ± 0.39, p = 0.46, **Figure 2D**), but no difference in C3 deposition was observed in the cortex or hippocampus (**Supplementary Figure 1**). Moreover, there were no differences in IgG or albumin deposition in the cortex, hippocampus, or CP between MRL/lpr LCN2-KO and MRL/lpr mice (LCN2-KO (n = 4) vs. MRL/lpr (n = 8); **Figures 2E, F**, **Supplementary Figure 1**). These results suggest that LCN2 deficiency decreases immune cell infiltrates in the CP of MRL/lpr mice, but not BBB leakage.

**Figure 3:**
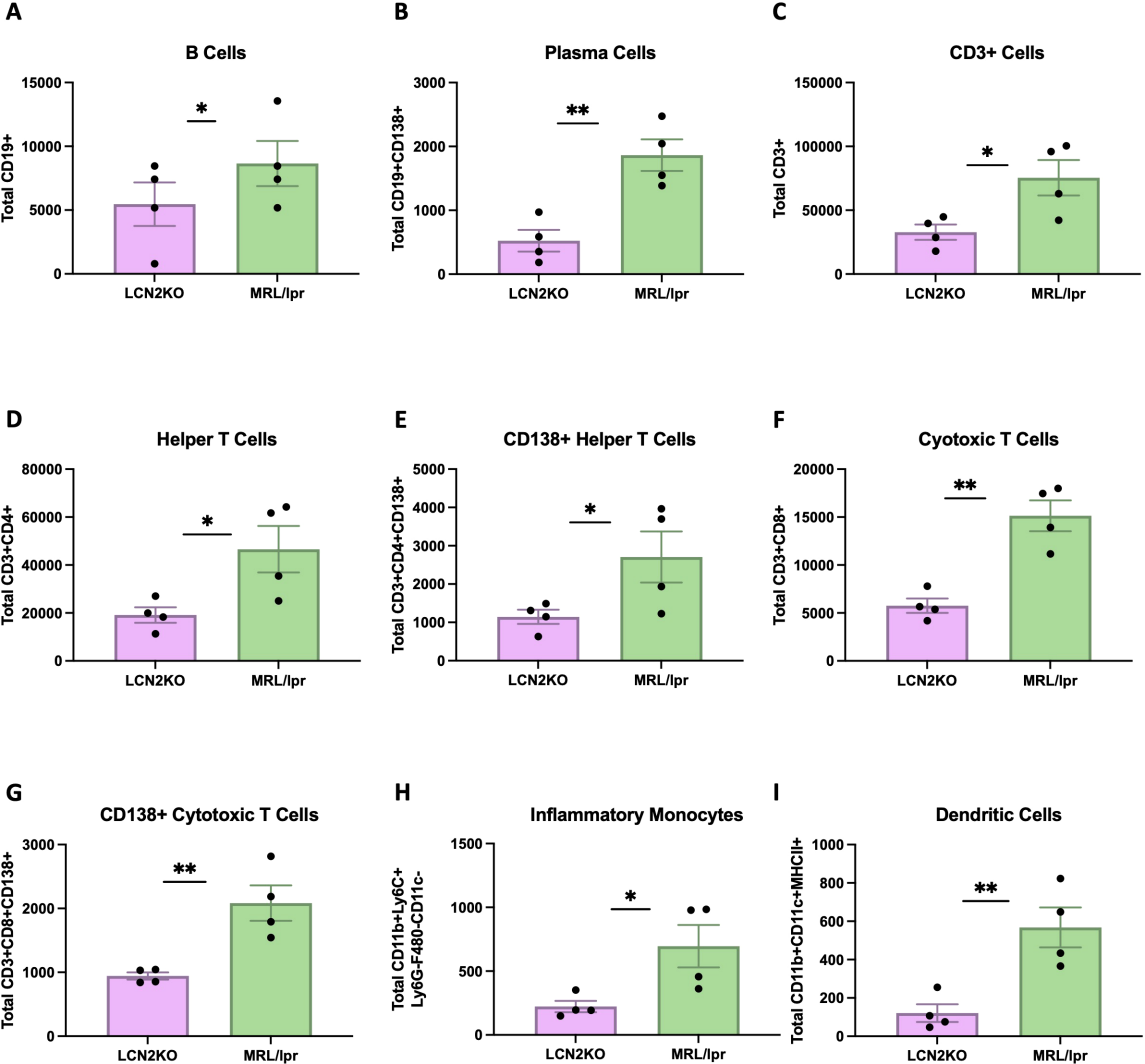
LCN2 deficiency decreases immune cell counts in MRL/lpr brains. Cell counts were calculated per whole brain. **(A-I)** B cells, plasma cells, total T cells, helper T cells (CD4+), CD138+ helper T cells, cytotoxic T cells, CD138+ cytotoxic T cells, inflammatory monocytes, and dendritic cells respectively were all significantly decreased in the brains of LCN2-deficient as compared to wild-type (LCN2 sufficient) MRL/lpr mice. For all panels: *p<0.05 and **p<0.01.

We sought to accurately quantify immune cell infiltration into the brain, and developed a multiparameter flow cytometry panel to carefully identify multiple immune cell subsets in whole brains of LCN2-KO and MRL/lpr mice. The cell types were characterized using the expression of markers as detailed in the provided table (**Supplementary Table 2**). We found that the total number of brain B cells was decreased in LCN2-KO mice (LCN2-KO (n = 3) vs. MRL/lpr (n = 3), 2637 ± 650 vs 8654 ± 1770, p = 0.02, **Figure 3A**). LCN2-KO mice additionally showed decreased plasma cell counts infiltrating the brain (LCN2-KO (n = 3) vs MRL/lpr (n = 3), 524 ± 170 vs 1863 ± 247, p = 0.04, **Figure 3B**). T cells have been shown to play an important role in NPSLE development (21). We quantified the total number of T cell infiltrates as well as some key subsets and found that T cells were significantly reduced in LCN2-KO brains (LCN2-KO (n = 3) vs MRL/lpr (n = 3), 32,814 ± 5,974 vs 75,380 ± 13,879, p =A 0.03, **Figure 3C**). Helper T cells were one of the subsets reduced by LCN2 deficiency (LCN2-KO (n = 3) vs MRL/lpr (n = 3), 19,144 ± 3,224 vs 46,595 ± 9,696, p = 0.04, **Figure 3D**). We observed expression of CD138 in T cell subsets and found that CD138+ helper T cells were also decreased in LCN2-KO brains (LCN2-KO (n = 3) vs MRL/lpr (n = 3), 1,144 ± 185 vs 2,796 ± 668, p = 0.04, **Figure 3E**).Cytotoxic T cells and CD138+ cytotoxic T cell infiltrates were reduced in LCN2-KO brains (LCN2-KO (n = 3) vs MRL/lpr (n = 3), 5,753 ± 752 vs 15,135 ± 1,603, p = 0.002; 943 ± 55 vs 2,085 ± 277, p = 0.007, **Figures 3F, G**). As monocytes contribute to organ damage in SLE, we also quantified the number of monocytes. We found a reduction in inflammatory monocytes as well as dendritic cells with LCN2 deficiency (LCN2-KO (n = 3) vs MRL/lpr (n = 3), 223 ± 44 vs 696 ± 166, p = 0.03; 121 ± 46 vs 568 ± 104, p = 0.007, **Figures 3H, I**). We quantified other cell subsets, including Tregs, Th17 cells, macrophages, and neutrophils, and did not find any differences in brain infiltration between MRL/lpr mice and LCN2-deficient mice (**Supplementary Figure 2**). Our results show that LCN2 deficiency differentially decreases immune cell infiltration into the brains of MRL/lpr mice.

### 3.3 Systemic autoimmunity is unaffected by LCN2 deficiency

**Figure 4:**
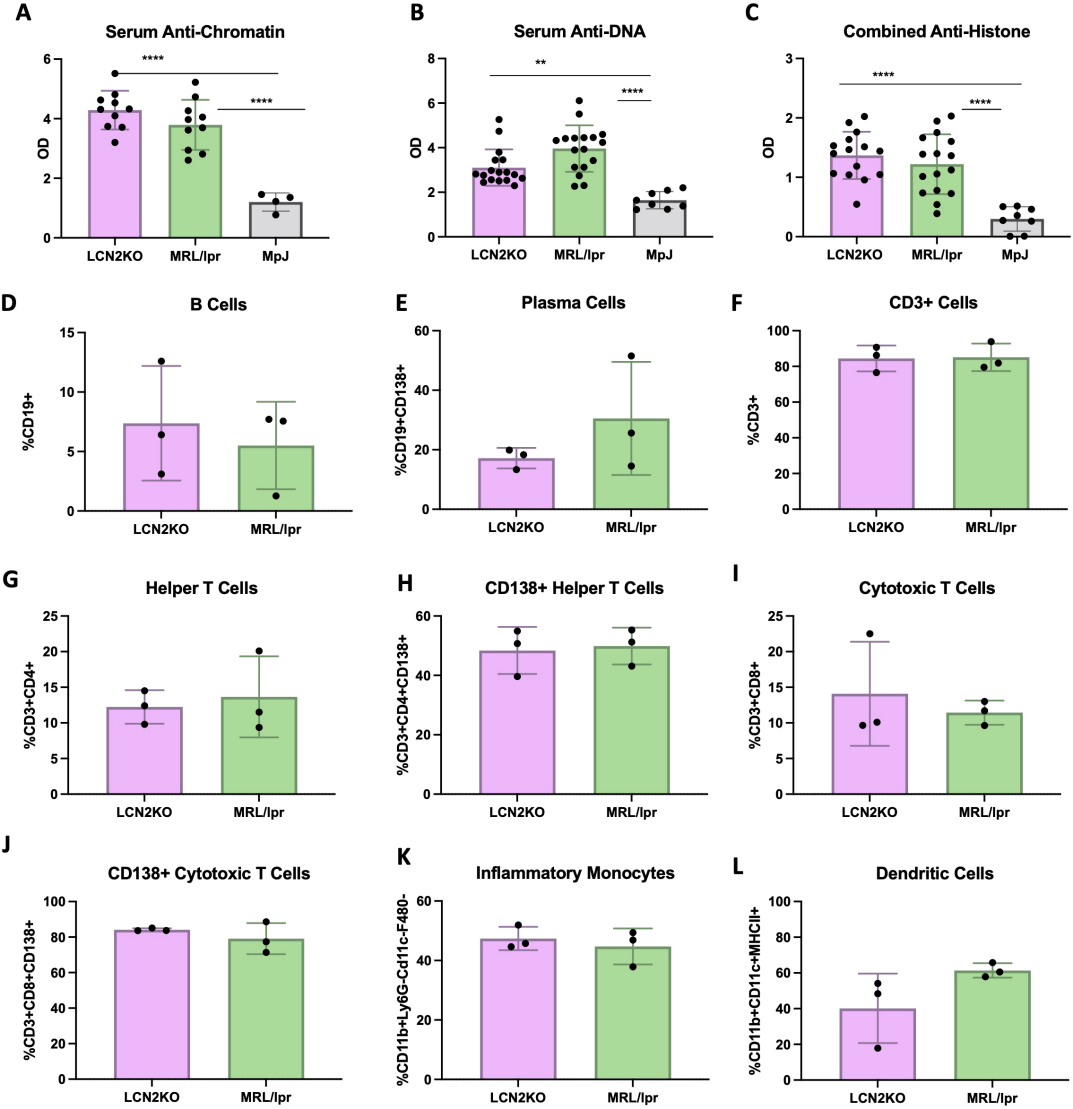
LCN2 does not mitigate systemic autoimmunity and inflammation. LCN2 has no effect on serum autoantibodies including **(A–C)** anti-chromatin IgG, anti-DNA IgG, and anti-histone IgG. No effect is seen in circulating PBMC numbers including **(D-L)** B cells, plasma cells, T cells, helper T cells, cytotoxic T cells, CD138+ helper T cells, cytotoxic T cells, CD138+ cytotoxic T cells, inflammatory monocytes, and dendritic cells respectively. For all panels: **p<0.01 and ****p<0.0001.

One advantage of the MRL/lpr strain as a lupus model is its ability to recapitulate key features of human disease, including nephritis, as well as hypergammaglobulinemia and increased concentrations of circulating autoantibodies directed against nuclear antigens. Kidney disease was semi-quantitatively monitored by measuring proteinuria using Uristix, but we found no differences in kidney disease between MRL/lpr and LCN2-KO mice (**Supplementary Figure 3A**). To evaluate the possible effects of LCN2 deficiency on systemic autoimmunity, we measured the levels of anti-chromatin, anti-histone, and anti-dsDNA antibodies in the serum of MRL/lpr LCN2-KO and MRL/lpr mice compared to those in the MRL/Mpj strain. While MRL/lpr LCN2-KO mice had significantly higher levels of anti-dsDNA, anti-chromatin, or anti-histone antibodies than MRL/Mpj mice, these were not different from those in the parent LCN2 sufficient strain (**Figures 4A–C**). Similarly, the total IgG concentration was not regulated by the presence of LCN2 (**Supplementary Figure 3B**). These results showed that despite improving behavioral outcomes, LCN2 deficiency did not improve systemic autoimmunity in MRL/lpr mice.

**Figure 5:**
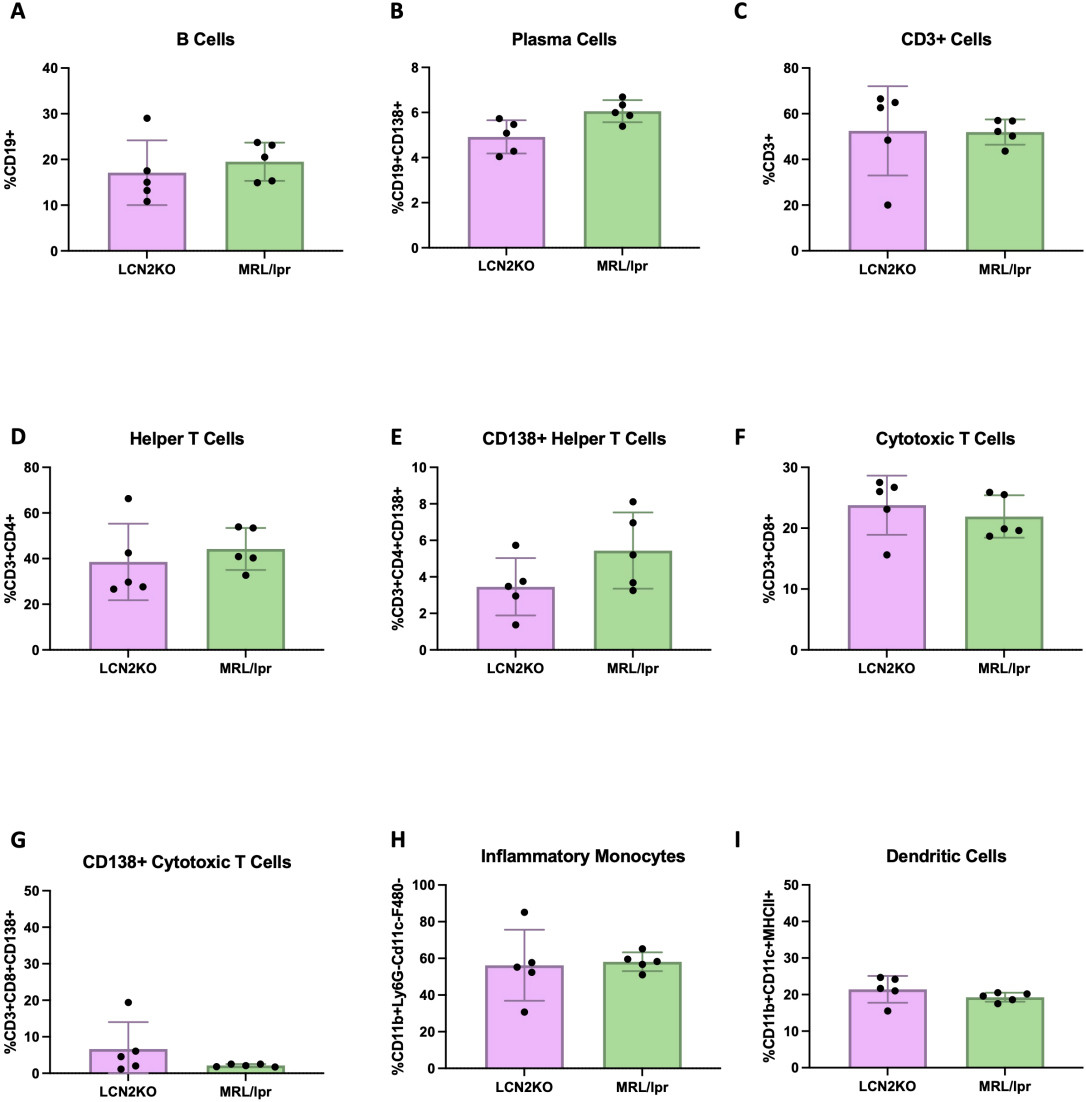
LCN2 deficiency does not affect spleen cell subsets. The number of immune cells in the spleen was not different between MRL/lpr and LCN2-KO MRL/lpr mice. **(A-I)** B cells, plasma cells, T cells, helper T cells, cytotoxic T cells, CD138+ helper T cells, cytotoxic T cells, CD138+ cytotoxic T cells, inflammatory monocytes, and dendritic cells, respectively. None of the panels depict significant differences.

Unchanged IgG and anti-nuclear antibodies suggested that there was no decrease in systemic inflammation in LCN2 deficient MRL/lpr mice. To support this conclusion, we quantified the levels of immune cells in PBMCs and secondary lymphoid organs of LCN2-KO and MRL/lpr mice. Flow cytometry was used to quantify the proportion of immune cells in PBMCs, lymph nodes, and spleen. Unlike in the brain, we found that LCN2 deficiency did not affect circulating immune cell subset distribution in PBMCs (**Figures 4D–L**). We quantified the same 18 cell types in the spleen and found that no cell subsets were affected by LCN2 deficiency (**Figures 5A–I**). Similarly, no differences in immune cellular distribution were present in the lymph nodes of LCN2-KO mice compared with that in MRL/lpr mice (data not shown). These results suggest that the effects of LCN2 are organ specific, since deficiency has no effect on manifestations of systemic autoimmunity in MRL/lpr mice.

### 3.4 LCN2 deficiency improves cutaneous disease in MRL/lpr mice

**Figure 6:**
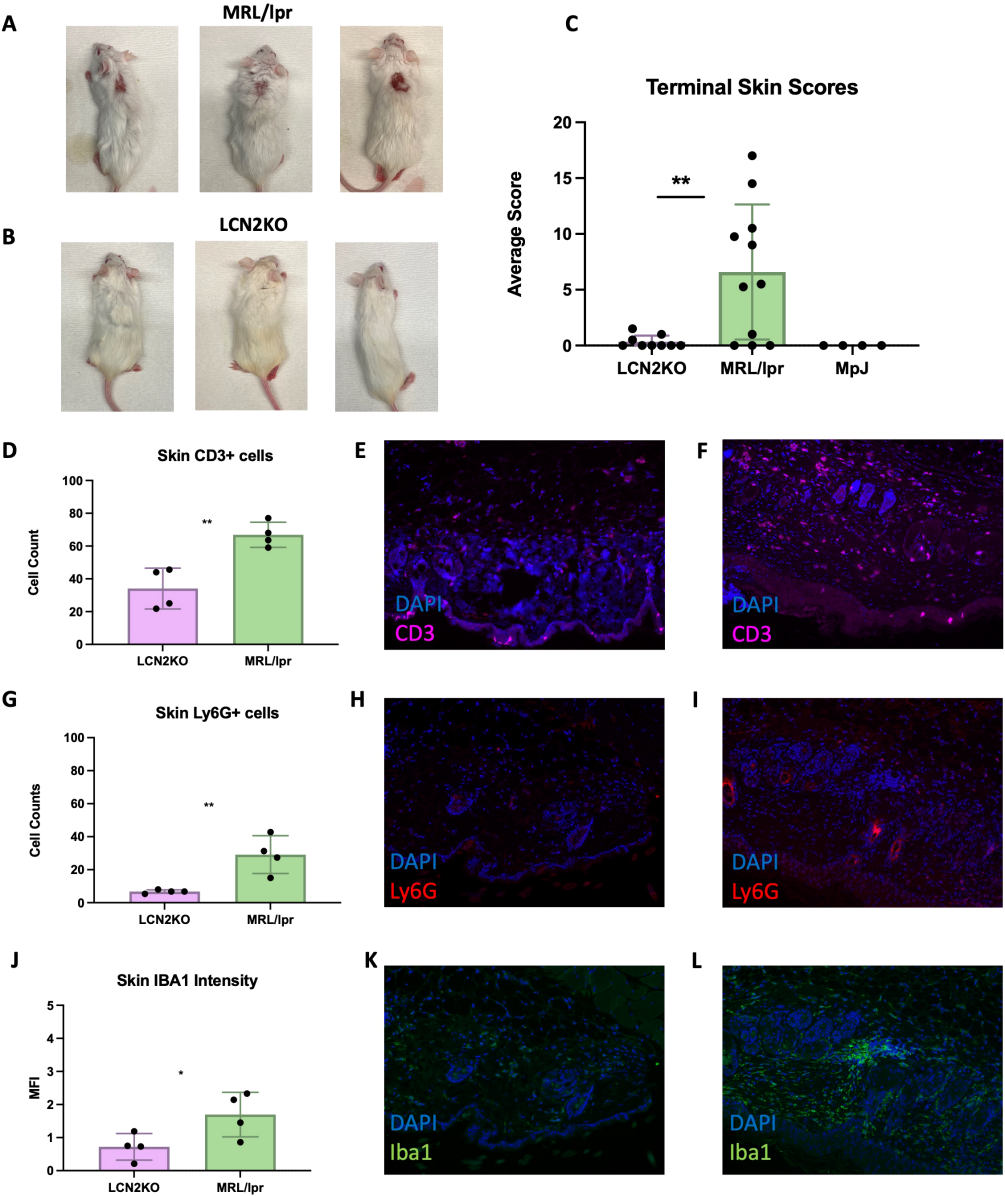
LCN2 deficiency improves cutaneous disease in MRL/lpr mice. **(A)** A representative image of lesional skin on MRL/lpr mice. Erythema, alopecia, and skin thickening are apparent. **(B)** shows a markedly improved skin phenotype in LCN2-KO mice. **(C)** Quantitative skin scores (recorded blindly) confirmed the significant improvement in skin disease in LCN2-KO mice. **(D)** T cells were decreased in the skin of LCN2-deficient mice. **(E)** Representative IF images of skin from LCN2-KO MRL/lpr stained for T cells. **(F)** Representative IF image of T cells in MRL/lpr lesional skin. **(G)** Neutrophils were decreased in the skin of LCN2-KO mice. **(H)** Representative image of cutaneous neutrophils in LCN2-KO mice. **(I)** Representative IF image of cutaneous neutrophils in MRL/lpr mice. **(J)** LCN2 deficiency reduced skin macrophage infiltrates. **(K)** Representative IF image of LCN2-KO skin macrophage infiltrates. **(L)** MRL/lpr skin macrophage infiltrates. For all panels: *p<0.05, **p<0.01.

Another significant advantage of the MRL/lpr strain compared to other murine lupus models is the development of a chronic erythematous facial rash with hair loss extending to the back, analogous to skin involvement in patients with lupus or cutaneous lupus erythematosus. To study the effect of LCN2 on the development of other types of classic lupus organ involvement besides the brain, MRL/lpr and MRL/lpr LCN2-KO mice were evaluated for the extent and severity of skin disease using validated scores for alopecia, erythema, and skin thickening of the back and face (27). At 18 weeks of age, MRL/lpr LCN2-KO mice showed a significant improvement in skin disease compared with that of MRL/lpr mice (**Figures 6A–C**). We quantified immune cell infiltrates in the lesional skin by staining samples for T cells (CD3+), neutrophils (Ly6G+), and macrophages (Iba1). We found a significant reduction in CD3+ T cells in MRL/lpr LCN2-KO mice (LCN2-KO (n = 4) vs MRL/lpr (n = 4), 34 ± 6 vs 67 ± 4, p = 0.004, **Figures 6D–F**). Neutrophils, another cell type associated with cutaneous disease in SLE, were also reduced in LCN2-deficient mice compared to MRL/lpr mice (LCN2-KO (n = 4) vs MRL/lpr (n = 4), 7 ± 1 vs 29 ± 6, p = 0.003, **Figures 6G–I**). The third cell type evaluated in the lesional skin samples was macrophages, whose aberrant activation leads to tissue damage in multiple SLE organs (including the skin). Similar to what was found for T cells and neutrophils, MRL/lpr LCN2-KO mice showed significantly reduced expression of the macrophage marker IBA1 in lesional skin of MRL/lpr LCN2-KO mice (MRL/lpr LCN2-KO (n = 4) vs MRL/lpr (n = 4), 1 ± 0.2 vs 2 ± 0.3, p = 0.05, **Figures 6J–L**).

### 3.5 LCN2 2 activates astrocytes in an autocrine manner

In other neurological contexts, activated astrocytes have been shown to produce LCN2 with inflammatory effects (15, 33). To measure astrocyte activation, we quantified the brain expression of glial fibrillary acidic protein (GFAP). We observed decreased GFAP expression in the hippocampus of LCN2-KO mice compared to wildtype (**Supplementary Figure 4**). We then studied the source of LCN2 production in MRL/lpr mice by evaluating LCN2 expression in the different brain cell types. Following LPS injection systemically, we quantified the number of microglia or astrocytes co-stained with LCN2 using IF staining. A higher number of GFAP-positive cells, or astrocytes, were co-stained with LCN2 than IBA1+ cells, or microglia (**Supplementary Figure 5**). These results suggest that in MRL/lpr mice, LCN2 is produced by astrocytes in response to an inflammatory insult.

**Figure 7:**
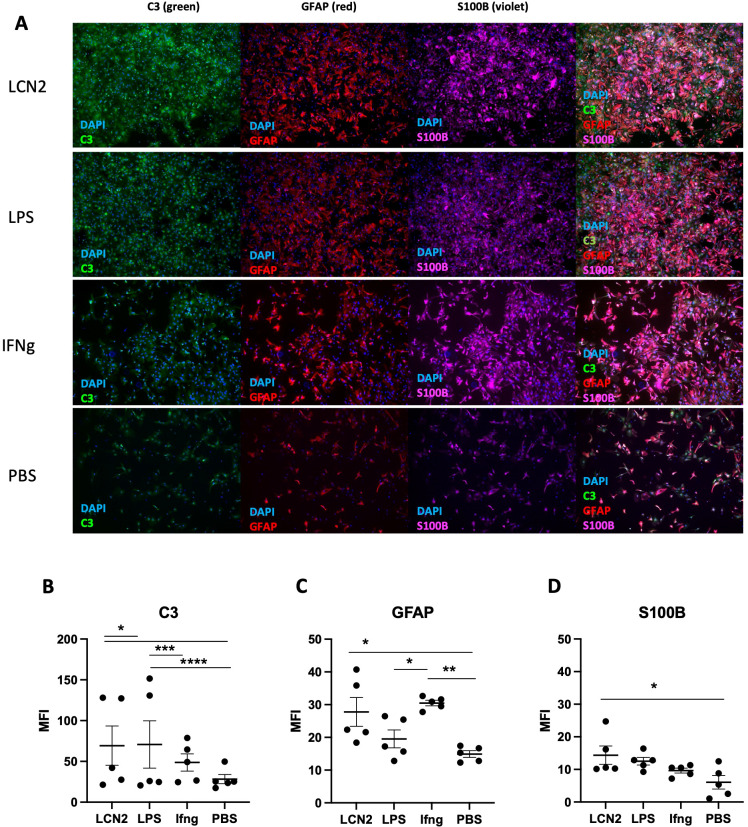
LCN2 promotes an inflammatory astrocyte phenotype. Cortical astrocytes isolated from newborn MRL/lpr pups were evaluated by immunofluorescence for the presence of activation markers following different inflammatory stimuli. LCN2 was added at a concentration of 25 μg/mL, LPS at a concentration of 100 ng/mL, and IFNγ was added at a concentration of 1 μg/mL. Treatment duration for each condition was 10 hours. **(A)** Representative images of treated astrocytes following each stimulus. Vertically listed are the different treatment conditions, while horizontally listed are the different activation markers being measured in each column. The last column in this panel shows the overlay of all 3 markers. **(B)** C3 protein expression is induced in LCN2 treated primary astrocytes. **(C)** LCN2 induces GFAP expression in primary astrocytes. **(D)** S100B, another astrocytic inflammatory marker, is also increased upon LCN2 exposure. For all panels: *p<0.05, **p<0.01, ***p<0.001, and ****p<0.0001.

To determine whether LCN2 activates astrocytes in MRL/lpr mice, we isolated cortical astrocytes from newborn MRL/lpr mice (32) and treated them with LCN2. Activated astrocytes upregulate C3, a complement protein (34). In addition, activated astrocytes are characterized by an increase in the cytoskeletal proteins GFAP and S100B (35, 36). Indeed, we found that astrocytes treated with LCN2 showed significant increases in C3, GFAP, and S100B levels compared with control astrocytes treated with PBS (**Figure 7A**)(LCN2 vs PBS (n = 5 in each group): C3: 70 ± 23 vs 30 ± 6, p = 0.04 (**Figure 7B**); GFAP: 28 ± 5 vs 15 ± 2, p = 0.04 (**Figure 7C**); S100B: 15 ± 3 vs 7 ± 2, p = 0.03 (**Figure 7D**). Altogether, these results suggest that astrocytes in MRL/lpr mice can both produce LCN2 and be activated by an increase in LCN2, suggesting a potential autocrine mechanism of action for LCN2 in the astrocytes of MRL/lpr mice.

## 4 Discussion

### 4.1 LCN2 modulates behavioral outcomes in NPSLE

The effect of LCN2 on the CNS is highly context-dependent; it has been implicated in anxiety, depression, and memory dysfunction, and exacerbates or attenuates CNS disease depending on the disease model (33). We therefore wanted to examine the role of LCN2 in the gold-standard model of NPSLE in MRL/lpr mice. In this study, we found that LCN2 deficiency improved behavior and decreased astrocyte activation and brain infiltration of immune cells in MRL/lpr mice. Cutaneous disease was also ameliorated with decreased skin lesion severity and immune cell infiltration. These improvements were localized to the brain and skin, since no differences were observed in systemic disease manifestations.

In our model, we observed an improvement in spatial memory, but not in recognition memory, in LCN2-deficient mice. The hippocampus is involved in depression and spatial memory (37). Olson et al. confirmed through their model of cachexia that LCN2 contributes to spatial, but not pattern recognition memory. They further determined that this improvement was attributable to hippocampal neuronal dysfunction caused by the acute and chronic expression of LCN2 in the CNS (38). We also observed a decrease in astrocyte activation in the hippocampus of LCN2-KO mice, supporting an effect of LCN2 in the lupus hippocampus. While our data indicate that LCN2 affects hippocampal processes, it is important to consider whether this improvement is directly due to LCN2 or an overall reduction in CNS inflammation. Synapse formation and neuronal function have not been well-studied in NPSLE mice, although previous studies have focused on the effects of immune cell infiltration and inflammation. Evaluating the specific effects of inflammation at the synaptic and neuronal levels would help to clearly determine how specific brain regions are affected.

LCN2 is typically secreted by microglia or astrocytes under inflammatory conditions (12, 13), therefore, we confirmed that at least locally astrocytes were the main source of LCN2 production in MRL/lpr mouse brains, and that LCN2 deficiency decreased the activation of hippocampal astrocytes. This finding, combined with the effects of LCN2 on spatial memory and depression, suggests that LCN2 modulates hippocampal function in MRL/lpr mice and is an important mediator of the neurobehavioral phenotype in this strain.

Unlike patients with lupus, MRL/lpr mice do not experience discrete flares or disease exacerbations but rather progressive disease; consequently, quantifying real-time LCN2 expression is challenging due to lower LCN2 expression during chronic inflammation. Therefore, we injected LPS as a discrete inflammatory stimulus to mimic a disease flare to determine the source cell type for LCN2 expression. Developing an organoid model to study astrocytic LCN2 production during chronic inflammation might be useful for evaluating LCN2 production at a higher resolution. Although brain organoids cannot capture the total context of NPSLE, they are useful for observing how glial cells and neurons interact, which is relevant to neuroinflammation (39).

### 4.2 LCN2 deficiency reduces immune cell infiltrates in the brain, but not in systemic disease

We have previously described markedly increased CP infiltrates in the brains of MRL/lpr mice (21, 30, 40). LCN2-KO mice showed decreased CP infiltrate scores, whereas no differences were identified in cellular proliferation or apoptosis, as reflected by Ki67 and TUNEL staining respectively (data not shown). When characterizing the cell types in the brain most affected by LCN2, we found a reduction in the number of several key immune cell subsets, including T cells, B cells, and inflammatory monocytes, but not in neutrophils or macrophages. Consistent with our findings, a separate study investigating the effects of LCN2 on immune cell trafficking into the CNS following intracerebroventricular LCN2 injection showed an increase in T and B cells, but not neutrophils, suggesting that LCN2 differentially recruits specific cell types to the CNS (38). Our results support previous findings showing that the contribution of T and B cells to NPSLE is a major driver of neuropsychiatric diseases in MRL/lpr mice (30, 41). Processing brains for flow cytometric analysis compromises cell viability, and immune cell counts are typically quite low in mouse brains; therefore, we chose to analyze whole-brain samples and cannot determine at this time if the differences in cellular subsets found were region specific. In future studies it would be useful to determine (by immunohistochemistry or flow cytometry) whether these differences in immune cell distribution were consistent between multiple brain regions or specific to one or more. Either way, our comprehensive flow cytometry panel did provide a useful tool to quantify many cell subsets despite relatively limited sample availability.

One controversial topic in the understanding of the mechanisms of NPSLE is the postulated involvement of BBB disruption in the recruitment of immune cells to the CNS. Studies have supported the involvement of BBB disruption in NPSLE (40–43). In the present study, we found that LCN2 had no effect on BBB disruption, suggesting that the reduction in immune cell trafficking into the brain in LCN2-KO MRL/lpr mice was not mediated through a generalized effect on BBB permeability. BBB permeability is another facet of NPSLE that can also be more carefully studied using organoid models.

Notably, while LCN2 deficiency improved behavioral outcomes and immune cell recruitment to the CNS in MRL/lpr mice, it did not have a discernible effect on systemic disease. Anti-nuclear antibody levels, splenomegaly, lymph node size, and serum IgG levels were not affected by LCN2 deficiency. Moreover, there were no significant variations in the distribution of different cell types in the PBMCs, spleens, and lymph nodes of LCN2-KO mice compared to those in wild-type MRL/lpr mice, suggesting that LCN2 had a localized effect on the recruitment of immune cells to the brain, rather than modulating brain infiltration by ameliorating systemic inflammation or immune cell development. While some studies have identified LCN2 as a potential biomarker for human lupus nephritis (44–46), to the best of our knowledge, the only previous study on the effect of LCN2 in MRL/lpr mice involved the injection of anti-LCN2 antibodies or LCN2 recombinant protein and examining the effects on renal disease in this strain (47). In contrast to what might be expected from this report by Chen et al. (47), we found that global knockout of LCN2 in MRL/lpr mice did not have a mitigating effect on nephritis, which may be due to the development of alternative inflammatory pathways when the gene was constitutively knocked out.

### 4.3 LCN2 deficiency improves cutaneous lupus in MRL/lpr mice

An unexpected finding was the remarkable improvement in cutaneous disease in LCN2-KO MRL/lpr mice. The recruitment of immune cells that drive cutaneous disease is also dramatically reduced in lesional skin. LCN2 has also been implicated in other autoimmune skin diseases, including acute skin inflammation (48). Our results showed a reduction in neutrophils, macrophages, and T cells in inflamed skin, recapitulating a study in psoriasis showing LCN2-dependent skin infiltration (49). It has been suggested that LCN2 recruited immune cells to sites of cutaneous inflammation by augmenting Th17 responses (49, 50). Future studies investigating the role of LCN2 in MRL/lpr cutaneous disease should aim to further characterize immune cell infiltrates and quantify the cytokines most affected by LCN2.

The localized effect of LCN2 deficiency in MRL/lpr mice was surprising, as cutaneous and neuropsychiatric diseases improved but systemic and kidney disease were unaffected. This could be due to a discrepancy in the local immune signaling pathways, which are regulated in a tissue-specific manner. B and T cells, but not neutrophils, were reduced in the skin and brain upon LCN2 deficiency, suggesting both tissue and cell type specific regulation. Another potential contributor to this observed effect could be differential developmental patterns of skin and brain tissues (e.g. the localization of immune cells occurring during embryonic development).

Another potential limitation of our analysis concerns the use of primary astrocytes from P0 pups. While *in vitro* studies help characterize the genotype-intrinsic effects on activation and phenotype, they may not recapitulate the full spectrum of inflammatory mediators produced in SLE. Additionally, because NPSLE symptoms appear as early as 6–8 weeks in MRL/lpr mice (51), the results obtained from newborn mice may not be as informative as those obtained from adult mice. Furthermore, while our LPS experiments elucidated the source of LCN2 in an inflammatory environment, it would be useful to quantify baseline levels of brain LCN2 expression in MRL/lpr mice using more sensitive methods. Finally, while a constitutively LCN2-deficient strain highlights the importance of LCN2 in SLE, cell-specific knockouts would help further specify the cell types or signaling pathways through which LCN2 might contribute to disease in specific tissues.

Previously, we investigated the role of LCN2 in the B6.SLE1.3 mouse model. In the present study, we examined the effects of LCN2 depletion in MRL/lpr mice, a more widely used murine SLE model with much more pronounced NPSLE involvement. In the B6.SLE1.3 strain, we found that LCN2 deficiency improved behavioral and emotional deficits (17). In LCN2-deficient MRL/lpr mice, we observed an improvement in depression-like behaviors and learned helplessness. This finding is consistent with those of previous studies in stroke and inflammatory bowel disease associated with depression and elevated LCN2 levels (52, 53).

Our study identified a localized and differentiated role of LCN2 in target organ injury in a robust spontaneous lupus model. Besides discovering a decrease in the activation of astrocytes in the hippocampus, cells which were also a source of LCN2 in the brain, we identified cell types whose recruitment to the brain was regulated by LCN2 although LCN2 deficiency did not affect the distribution of these cell types in the blood and secondary lymphoid organs. Taken together, these pre-clinical studies strongly support exploring the role of LCN2 in neuropsychiatric and skin disease associated with human SLE.

### 4.4 Significance of current study

We established that LCN2 is a driver of cutaneous and neuropsychiatric involvement, but not systemic disease, in the MRL/lpr mouse model. These results found in a more robust and representative murine model of NPSLE build upon our previous observations and further establish the role of LCN2 in NPSLE. The effects of LCN2 on immune cell recruitment into the brain and skin provide an important look into the possible mechanism of action of LCN2 in these common lupus manifestations. Since our results show improvements in tissue-specific infiltrates, but not the distribution of immune cells in the spleen or circulating PBMC, it is likely that LCN2 plays a role in immune cell recruitment, rather than development.

## Data Availability

The raw data supporting the conclusions of this article will be made available by the authors, without undue reservation.
