# Supplementary material for: Lipocalin-2 drives neuropsychiatric and cutaneous disease in MRL/lpr mice
Source: Front Immunol. 2024 Sep 27;15:1466868. doi: 10.3389/fimmu.2024.1466868 (PMC11466786; doi:10.3389/fimmu.2024.1466868)
Supplement: Supplementary file 1 [file DataSheet1.docx]

# Supplementary Table 1. Detailed list of antibodies used in panel for flow cytometry experiments.

#

| **Flow Cytometry Panel Details** | | | |
| --- | --- | --- | --- |
| **Marker** | **Clone** | **Fluorophore** | **Supplier** |
| CD8 | 53-6.7 | BUV395 | BD |
| CD45 | 30-F11 | Alexa Fluor 350 | R&D Systems |
| MHCII | M5/114.15.2 | BUV496 | BD |
| CD19 | 1D3 | BUV563 | BD |
| CD4 | GK1.5 | BUV737 | BD |
| CD11b | M1/70 | BUV805 | BD |
| CD56 | 809220 | BV421 | BD |
| Ly-6C | HK1.4 | Pacific Blue | BioLegend |
| CD11c | N418 | BV510 | BioLegend |
| F4/80 | CI:A3-1 | StarBright SBV570 | Bio-Rad |
| IL-17A/RatIgG1 | TC11-18H10/R3-34 | BV650 | BD |
| CD196(CCR6) | 29-2L17 | BV785 | BioLegend |
| CD138 | 281-2 | BB515 | BD |
| CD3 | 17A2 | Alexa Fluor 532 | Thermo |
| Ly6G | 1A8 | PerCP | BioLegend |
| FOXP3/Rat IgG2a | FJK-16s/eBR2a | PerCP-eFluor 710 | Thermo |
| CD30 | mCD30.1 | PE | Biolegend |
| B220 | RA3-6B2 | PE/Fire 700 | BioLegend |
| Viability | Fixable | Zombie NIR | BioLegend |
| NK1.1/CD161 | S17016D | APC/Fire 810 | BioLegend |

# Supplementary Table 2. Cell type characterization for flow cytometric quantification. Cell types analyzed were characterized using the markers listed in the table. The cells were gated as live cells, and doublets were removed.

| **Markers used to identify cell types** | |
| --- | --- |
| **Cell Type** | **Markers** |
| B cells | CD45+CD19+ |
| B cells (MHCII+) | CD45+CD19+MHCII+ |
| Plasma cells | CD45+CD11b+Ly6C+LyG6-F480-CD11c- |
| T cells | CD45+CD3+ |
| Cytotoxic T cells | CD45+CD3+CD8+ |
| Cytotoxic T cells (CD138+) | CD45+CD3+CD8+CD138+ |
| Helper T cells | CD45+CD3+CD4+ |
| Helper T cells (CD138+) | CD45+CD3+CD4+CD138+ |
| DNT (CD138+) | CD45+CD3+CD4-CD8-CD138+ |
| Double negative T cells (DNT) | CD45+CD3+CD4-CD8- |
| Regulatory T cells | CD45+CD3+CD4+FoxP3+ |
| Th17 cells | CD45+CD3+CD4+IL17+ |
| Natural killer T cells | CD45+CD3+NK1.1+CD56+ |
| Natural killer cells | CD45+CD3-NK1.1+CD56+ |
| Neutrophils | CD11b+Ly6G+Ly6C+ |
| Dendritic cells | CD45+CD11b+CD11c+MHCII+ |
| Inflammatory monocytes | CD45+CD11b+Ly6C+LyG6-F480-CD11c- |
| Macrophages | CD45+CD11b+Ly6G-Cd11c-F480+ |

# Supplementary Figure 1

#
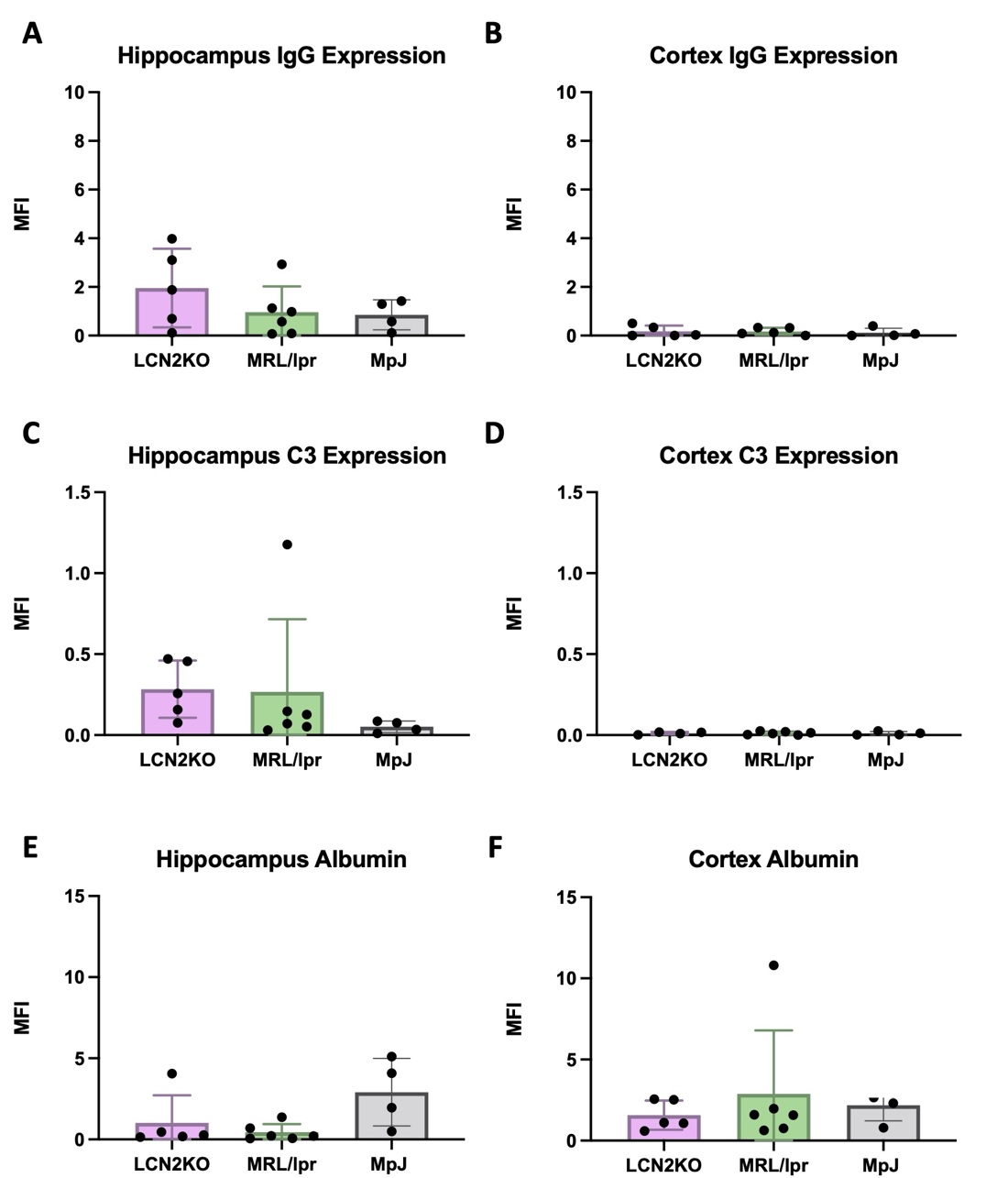


**Figure S1:** Staining of secreted factors in the hippocampus and cortex of MRL/lpr mice showed no differences upon LCN2 deficiency. **(A)** Hippocampal IgG expression was unaltered by LCN2 deficiency. **(B)** Cortical expression of IgG was unaffected by LCN2 deficiency. **(C)** Hippocampal C3 expression was not reduced by LCN2 deficiency. **(D)** Cortical C3 expression unaffected by LCN2 deficiency. **(E)** Hippocampal albumin was similar among all groups. **(F)** Cortical albumin levels were similar between LCN2KO and MRL/lpr mice.

#
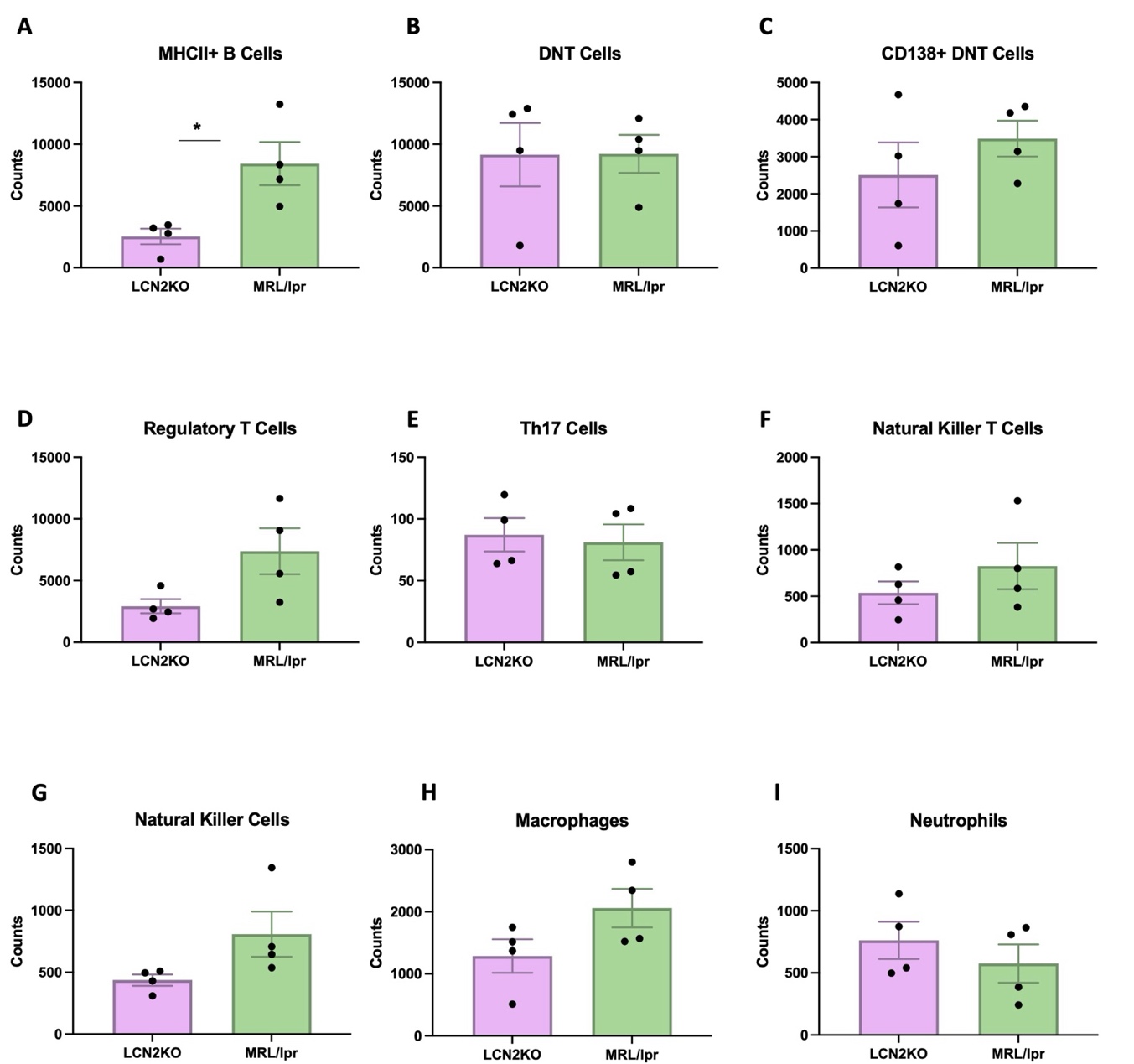
Supplementary Figure 2

**Figure S2:** Additional subsets quantified in brains showed no differences between LCN2 deficient and wildtype MRL/lpr strains. Cell counts were calculated per whole brain. **(A)** Consistent with our findings in B cell quantities, MHCII+ B cells were decreased in LCN2KO mice. **(B-E)** While some T cell subset counts were decreased in LCN2KO mice, other helper T cell subsets were unaffected by LCN2 deficiency. **(F,G)** Neither NK or NKT cells were decreased by LCN2 deficiency. **(H)** Resident macrophages in MRL/lpr brains were unaffected by LCN2 deficiency. **(I)** Neutrophils were present in similar quantities in the brains of LCN2KO and MRL/lpr mice.

## Supplementary Figure 3


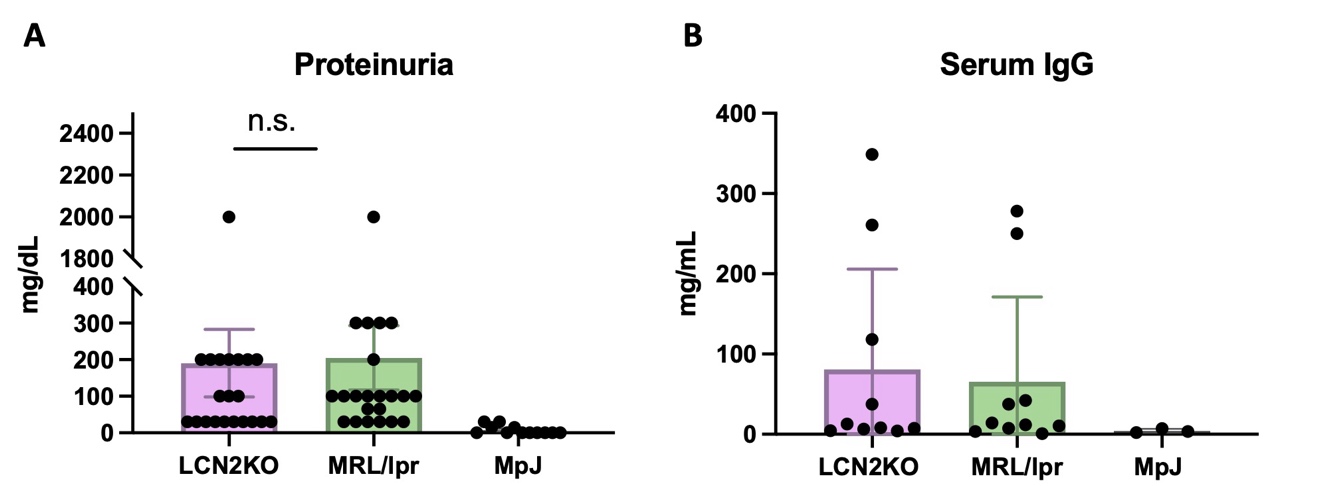


**Figure S3:** The effects of LCN2 deficiency did not affect systemic inflammation or kidney disease in MRL/lpr mice. **(A)** Proteinuria as measured semi-quantitatively by Uristix was unchanged in LCN2-deficient mice. **(B)** IgG titers were unchanged between MRL/lpr and LCN2 deficient mice. For all samples: n.s. = not significant.

## Supplementary Figure 4

##
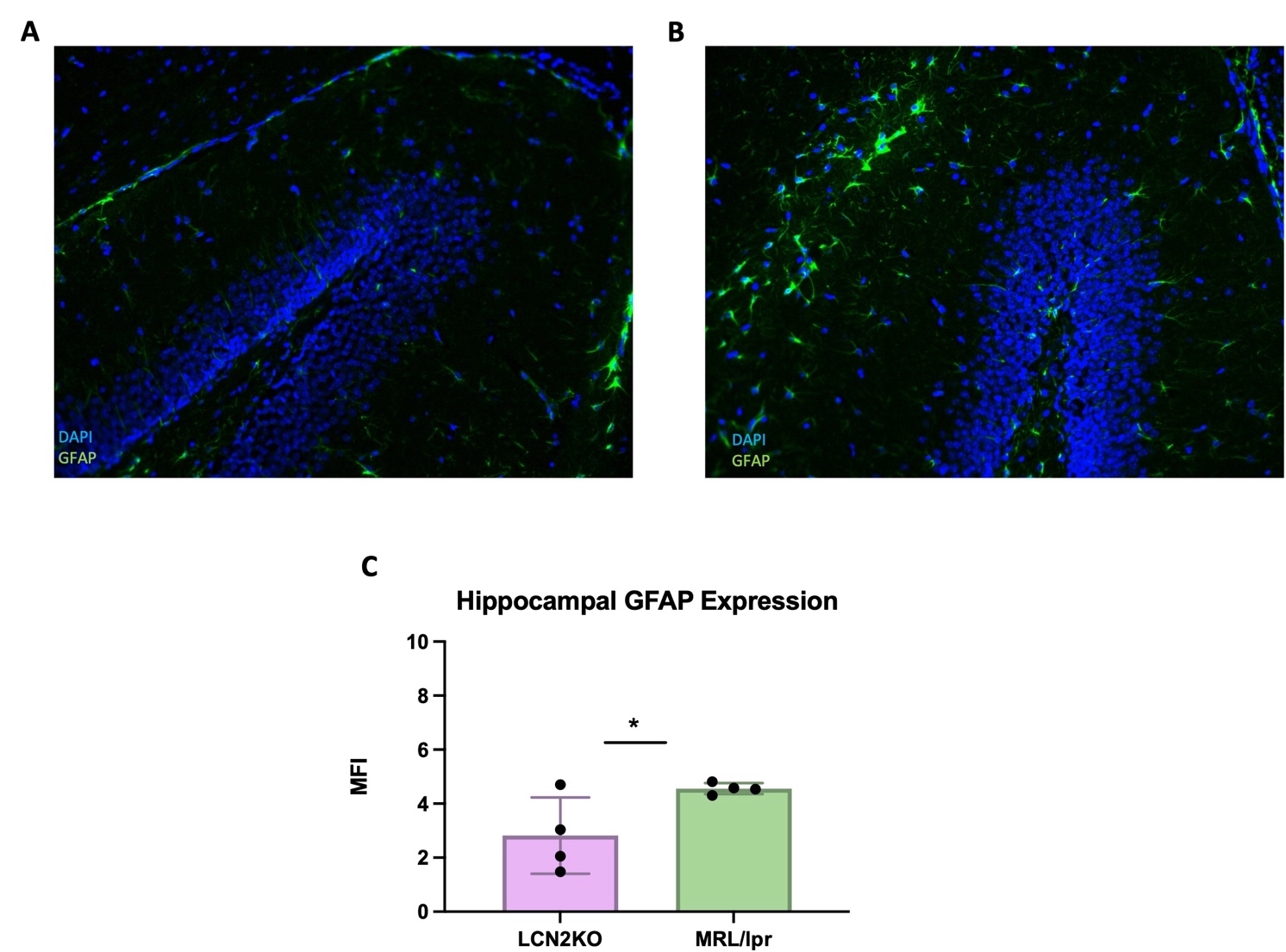


**Figure S4:** LCN2 deficiency decreased GFAP expression in the hippocampus of MRL/lpr mice. **(A)** Representative image of a stained LCN2-KO hippocampus. **(B)** Representative image of a stained MRL/lpr hippocampus. **(C)** Quantified mean fluorescence intensity (MFI) of hippocampal GFAP expression. For all samples: *p<0.05.

## Supplementary Figure 5


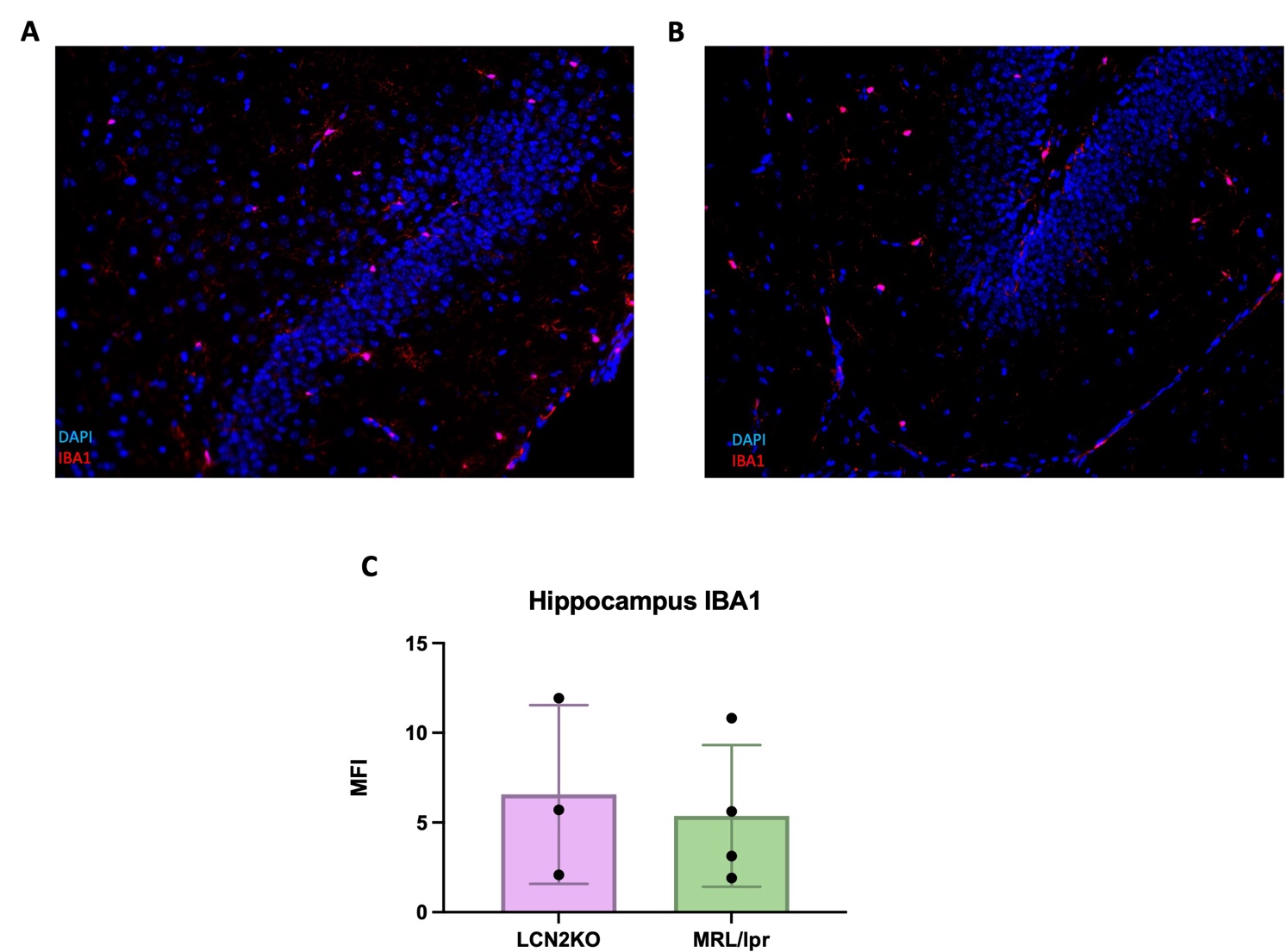


**Figure S5:** LCN2 deficiency has no effect on IBA1 expression in the hippocampus of MRL/lpr mice. **(A)** Representative image of a stained LCN2-KO hippocampus. **(B)** Representative image of a stained MRL/lpr hippocampus. **(C)** Quantified mean fluorescence intensity (MFI) of hippocampal IBA1 expression.

**Supplementary Material and Methods**

**Mice**

To generate a MRL/lpr strain deficient in LCN2, MRL/lpr mice were first crossed into the B6.LCN2KO strain. The progeny of this mating were then backcrossed with MRL/lpr mice for 10 generations, resulting in a congenic strain which is >99.9% genetically identical to the parent background strain. The selection of mice for breeding in each generation was based on the presence of the lpr mutation and LCN2 knockout, as determined by automated genotyping PCR performed by Transnetyx (Cordova, TN). The mutated, LCN2 deficient MRL/lpr strain demonstrated all the key phenotypic features of the MRL/lpr lupus prone strain (e.g. autoantibodies, hypergammaglobulinemia, and target organ involvement).

**
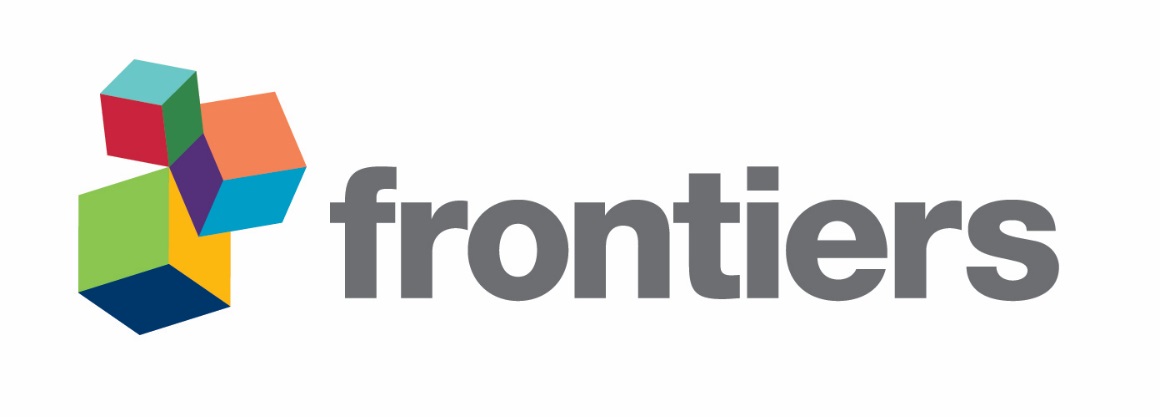
**
